# Chemical Composition and Insecticidal Activity against *Sitophilus zeamais* of the Essential Oils of *Artemisia capillaris* and *Artemisia mongolica*

**DOI:** 10.3390/molecules15042600

**Published:** 2010-04-12

**Authors:** Zhi Long Liu, Sha Sha Chu, Quan Ru Liu

**Affiliations:** 1 Department of Entomology, China Agricultural University, Haidian District, Beijing 100094, China; E-Mails: zhilongliu@cau.edu.cn (Z.L.L.); chushasha3421@126.com (S.S.C.); 2 College of Life Sciences, Beijing Normal University, Haidian District, Beijing 100875, China

**Keywords:** *Artemisia capillaris*, *Artemisia mongolica*, *Sitophilus zeamais*, fumigant, contact toxicity, essential oil

## Abstract

In our screening program for new agrochemicals from local wild plants, *Artemisia capillaris* and *A. mongolica* were found to possess insecticidal activity against the maize weevil, *Sitophilus zeamais*. The essential oils of aerial parts of the two plants were obtained by hydrodistillation and were investigated by GC and GC-MS. The main components of *A. capillaris* essential oil were 1,8-cineole (13.75%), germacrene D (10.41%), and camphor (8.57%). The main constituents of *A. mongolica* essential oil were α-pinene (12.68%), germacrene D (8.36%), and γ-terpinene (8.17%). Essential oils of *A. capillaris* and *A. mongolica* possess fumigant toxicity against *S. zeamais* adults with LC_50_ values of 5.31 and 7.35 mg/L respectively. The essential oils also show contact toxicity against *S. zeamais* adults with LD_50_ values of 105.95 and 87.92 μg/adult, respectively.

## 1. Introduction

Fumigation plays a very important role in insect pest elimination in stored products [1]. Plant essential oils and their components have been shown to possess potential for development as new fumigants and they may have advantages over conventional fumigants in terms of low mammalian toxicity, rapid degradation and local availability [2,3]. Essential oils derived from more than 75 plant species have been evaluated for fumigant toxicity against stored product insects so far [4].

Botanical pesticides have the advantage of providing novel modes of action against insects that can reduce the risk of cross-resistance as well as offering new leads for design of target-specific molecules [2,5]. During our screening program for new agrochemicals from local wild plants and Chinese medicinal herbs, the essential oils from aerial parts of *Artemisia capillaris* Thunb. and *A. mongolica* (Fish. et Bess.) Nakai (Family: Compositae) have been found to possess insecticidal activity towards the maize weevil, *Sitophilus zeamais* (Motsch). 

*Artemisia mongolica* is a species of perennial herbaceous rhizome plant of the Compositae family, used in Inner Mongolia as a substitute of the traditional medicinal herb Folium Artemisiae Argyi [6]. Most of the components isolated from the extracts of *A. mongolica* were terpenoids [7,8]. The chemical composition of *A. mongolica* essential oil was previously reported [9,10,11]. This plant was observed to be strongly resistant to insects and pathogens in the field [12].

*Artemisia capillaris* is a famous traditional Chinese medicinal herb used mainly as a choleretic, anti-inammatory, and diuretic agent in the treatment of epidemic hepatitis [6]. The chemical composition of the essential oils of *A. capillaris* has been widely studied [13,14,15,16,17,18,19]. This species was recorded to control cabbage white butterfly (*Pieris rapae*), cotton aphids (*Aphis gossypii*), cucurbit leaf beetle (*Aulacophora femoralis*) and other vegetable pests in China [20]. Several insect antifeedants were isolated and identified from the growing buds of *A. capillaris* [21,22,23,24]. Several essential oils derived from plant species of *Artemisia* have been evaluated for insecticidal activities against stored product insects [25,26,27,28,29,30]. However, no report on insecticidal activity of essential oils of *A. capillaris* and *A. mongolica* against stored product insects was available.

## 2. Results and Discussion

### 2.1. Chemical composition of the essential oils

The chemical compositions of the essential oils derived from the two *Artemisia* species are shown in Table 1 and Table 2. The main constituents of *A. mongolica* essential oil were 1,8-cineole (eucalyptol, 13.75%), germacrene D (10.41%), camphor (8.57%), artemisia ketone (6.96%) and calarene (5.62%). A total of 36 components were identified in the essential oil of *A. mongolica*, accounting for 95.82% of the total oil (Table 1). 

A total of 32 components were identified in the essential oil of *A. capillaris*, accounting for 95.21% of the total oil (Table 2) and the main components of the essential oil were β-pinene (12.68%), germacrene D (8.36%), γ-terpinene (8.17%), caryophyllene (7.66%) and 1,8-cineole (6.86%). The results were quite different from the previous reports. These differences might have been due to harvest time and local, climatic and seasonal factors as well as storage duration of medicinal herbs. For example, the essential oil of *A. capillaris* stalks and leaves collected in Japan contains capillene as a main component and limonene, β-pinene, β-elemene, β-caryophyllene, and α-humulene as minor components [14]. However, the essential oil of *A. capillaris* aerial parts collected in the fall from Qingdao, China contains capillene (31.41%), β-caryophyllene (21.64%), β-myrcene (8.84%) and limonene (6.03%) [15]. The main components of *A. capillaris* essential oil harvested in Korea were capillene (32.7%), β-pinene (9.4%), and β-caryophyllene (11.1%) [18]. Geographical variations in the chemical composition of *A. capillaris* essential oils obtained from four cities were observed [17,19] and seasonal variation in chemical constituents of *A. capillaris* was also observed [31]. Moreover, there were great variations of volatile components in different parts of *A. capillaris* [32].

### 2.2. Insecticidal activity

The essential oils of *A. mongolica* and *A. capillaris* showed contact toxicity against *S. zeamais* adults with LD_50_ values of 87.92 and 10.92 μg/adult, respectively (Table 3). Compared with the famous botanical insecticide, pyrethrum extract, the two essential oils were 20 times less active against the maize weevils because pyrethrum extract displayed a LD_50_ value of 4.29 μg/adult (Table 3). The essential oils of *A. mongolica* and *A. capillaris* also possess strong fumigant activity against *S. zeamais* adults with LC_50_ values of 7.35 and 5.31 mg/L, respectively (Table 3). The currently used grain fumigants, methyl bromide (MeBr) and phosphine were reported to have fumigant activity (24 h) against *S. zeamais* adults with LC_50_ values of 0.67 and 0.006 mg/L, respectively [33]. The two essential oils were 10 times less toxic to the maize weevil compared with the commercial fumigant MeBr. However, considering the currently used fumigants are synthetic insecticides, fumigant activity of the two essential oils is quite promising and they show potential to be developed as possible natural fumigants for control of stored product insects.

## 3. Experimental

### 3.1. Plant material

Fresh aerial parts (10 kg of leaves, stems and flowers) of *A. capillaris* and *A. mongolica* were harvested in August 2009 from Xiaolongmeng National Forest Park (Mentougou District, Beijing 102300). The aerial parts were air-dried for one week and ground to a powder. The species was identified, and the voucher specimens (BNU-liuzhilong-2009-08-29-013, BNU-liuzhilong-2009-08-29-014) were deposited at the Herbarium (BNU) of College of Life Sciences, Beijing Normal University.

### 3.2. Insects

The maize weevil, *S. zeamais* were obtained from laboratory cultures in the dark in incubators at 27–29 °C and 70-80% relative humidity. *S. zeamais* adults were reared on whole wheat at 12–13% moisture content. Unsexed adult weevils used in all the experiments were about 2 weeks old.

### 3.3. Essential oil distillation

The ground powder of *A. capillaris* and *A. mongolica* were subjected to hydrodistillation using a modified Clevenger-type apparatus for 6 h and extracted with *n*-hexane. Anhydrous sodium sulphate was used to remove water after extraction. Essential oils were stored in airtight containers in a refrigerator at 4 °C. The oil yields were 0.87% v/w and 0.75% v/w for *A. capillaris* and *A. mongolica*, respectively.

### 3.4. Gas chromatography and mass spectrometry

Gas chromatographic analysis was performed on an Agilent 6890N instrument equipped with a flame ionization detector and HP-5MS (30m × 0.25mm × 0.25μm) capillary column, while the essential oil components were identified on an Agilent Technologies 5973N mass spectrometer. The GC settings were as follows: the initial oven temperature was held at 60 °C for 1 min and ramped at 10 °C min^−1^ to 180 °C for 1 min, and then ramped at 20 °C min^−1^ to 280 °C for 15 min. The injector temperature was maintained at 270 °C. The samples (1 μL) were injected neat, with a split ratio of 1:10. The carrier gas was helium at flow rate of 1.0 mL min^−^^1^. Spectra were scanned from 20 to 550 m/z at 2 scans s^-1^. Most constituents were identified by gas chromatography by comparison of their retention indices with those of the literature or with those of authentic compounds available in our laboratories. The retention indices were determined in relation to a homologous series of *n*-alkanes (C_8_–C_24_) under the same operating conditions. Further identification was made by comparison of their mass spectra on both columns with those stored in NIST 05 and Wiley 275 libraries or with mass spectra from literature [39]. Component relative percentages were calculated based on GC peak areas without using correction factors. 

### 3.5. Fumigant toxicity

The fumigant activity of the two essential oils against *S. zeamais* adults was tested as described by Liu and Ho [33]. A serial dilution of the two essential oils (six concentrations) was prepared in *n*-hexane. A Whatman filter paper (diameter 2.0 cm) were each impregnated with 20 μL dilution, and then placed on the underside of the screw cap of a glass vial (diameter 2.5 cm, height 5.5 cm, volume 25 mL). The solvent was allowed to evaporate for 30 s before the cap was placed tightly on the glass vial, each of which contained 10 insects inside to form a sealed chamber. Preliminary experiments demonstrated that 30 s was sufficient for the evaporation of solvents. *n*-Hexane was used as a control. Five replicates were carried out for all treatments and controls, and they were incubated for 24 h. The insects were then transferred to clean vials with some culture media and returned to the incubator and observed daily for determination of end-point mortality, which was reached after one week. The experiments were repeated in three times. The LC_50_ values were calculated by using Probit analysis [34]. 

### 3.6. Contact toxicity

The contact toxicity of the two essential oils against *S. zeamais* adults was measured as described by Liu and Ho [33]. A serial dilution of the two essential oils (five concentrations) was prepared in *n*-hexane. Aliquots of 0.5 μL of the dilutions were applied topically to the dorsal thorax of the insects. Controls were determined using *n*-hexane. Both treated and control insects were then transferred to glass vials (10 insects/vial) with culture media and kept in incubators. Mortality of insects was observed daily until end-point mortality was reached one week after treatment. The experiments were repeated in three times. The LD_50_ values were calculated by using Probit analysis [34].

## 4. Conclusions

Based on mass screening, essential oils of *A. capillaris* and *A. mongolica* were examined for their insecticidal activity against maize weevils. The two essential oils possessed strong fumigant toxicity against the weevil adults, although they were 10 times less toxic to the maize weevil compared to commercial fumigant MeBr. The two essential oils also showed contact toxicity against maize weevils. These findings, considered together, suggest that the two essential oils show potential for development as natural fumigants for stored products.

## Figures and Tables

**Table 1:** Chemical composition of the essential oil of *Artemisia mongolica*.

Compounds	RI *	Relative content (%)
1,8-Cineole	1032	13.75
Artemisia ketone	1062	6.96
Thujone	1114	1.56
*p*-Menth-1-en-8-ol	1126	1.29
Camphor	1143	8.57
Borneol	1165	3.86
4-Terpineol	1179	2.36
Myrtenol	1196	0.37
(*S*)-Verbenone	1205	0.13
*cis*-Carveol	1226	0.06
D-Carvone	1242	0.14
Citrole/geraniol	1250	0.21
1,4-*p*-Menthadien-7-ol	1315	0.18
*p*-Vinylguaiacol	1323	0.27
γ-Pyronene	1345	0.70
α-Cubebene	1350	0.19
Eugenol	1356	0.99
Copaene	1374	1.12
β-Bourbonene	1385	2.27
β-Elemene	1388	1.37
β-Cubebene	1389	1.81
Calarene	1432	5.62
Germacrene D	1479	10.41
α-Zingiberene	1492	1.62
α-Muurolene	1498	1.08
1ξ,6ξ,7ξ-Cadina-4,9-diene	1502	0.73
α-Farnesene	1512	2.54
δ-Cadinene	1520	2.62
(*E*)-Nerolidol	1566	0.42
Spatulenol	1578	1.03
Davanone	1608	0.80
γ-Eudesmol	1621	1.10
tau-Cadinol	1640	0.48
α-Cadinol	1652	0.38
α-Bisabolol	1681	0.37
Phytol	2119	0.85
Total		95.82

* RI, retention index as determined on a HP-5MS column using the homologous series of *n*-hydrocarbons.

**Table 2:** Chemical composition of the essential oil of *Artemisia capillaris*.

Compounds	RI *	Relative content (%)
α-Pinene	931	0.72
β-Pinene	981	12.68
1,8-Cineole	1032	6.86
(*Z*)-Ocimene	1038	5.28
γ-Terpinene	1057	8.17
6-Acetophenone	1096	5.62
2-Methyl-6-methylene-1,7-octadien-3-one	1117	0.13
*cis*-*p*-Menth-2-en-1-ol	1126	0.12
4-Terpineol	1179	4.63
*p*-Menth-1-en-8-ol	1182	0.97
Citronellol	1213	0.30
*p*-Vinylguaiacol	1323	0.63
γ-Pyronene	1345	3.32
Eugenol	1356	3.12
Copaene	1377	0.90
β-Cubebene	1382	1.32
Diisopropenyl methylvinyl cyclohexane	1397	1.47
α-Cedrene	1409	4.64
Caryophyllene	1420	7.66
β-Farnesene	1438	5.71
Germacrene D	1479	8.36
Eremophilene	1489	0.97
Bicyclogermacrene	1494	3.67
δ-Cadinene	1520	2.08
β-Sesquiphellandrene	1524	4.21
*trans*-Nerolidol	1564	1.67
Longicamphenylone	1569	0.25
Spathulenol	1578	3.48
Globulol	1587	1.73
*epi*-α-Muurolol	1644	2.37
α-Cadinol	1652	2.21
Phytol	2119	0.65
Total		95.21

* RI, retention index as determined on a HP-5MS column using the homologous series of *n*-hydrocarbons.

**Table 3:** Insecticidal activity of the essential oils of *Artemisia capillaris* and *A. mongolica* against *Sitophilus zeamais* adults.

Essential oil	Contact toxicity (7 d)		Fumigant toxicity (7 d)	
	LD_50_ (μg/adult)	95% confidence limits	LC_50_ (mg/L)	95% confidence limits
*A. capillaria*	105.92	100.32-111.91	5.31	4.88-5.77
*A. mongolica*	87.92	80.57-95.69	7.35	6.60-8.27
Pyrethrum extract	4.29*			
MeBr	-	-	0.67**	-
Phosphine	-	-	0.006**	-

* data from Liu *et al*. [38]; ** data from Liu and Ho [35].
